# Epidemiology of Injuries in Elite Female Ice Hockey Players Allowed Body Checking: A Two‐Year Prospective Cohort Study

**DOI:** 10.1002/ejsc.70245

**Published:** 2026-08-27

**Authors:** Amanda Lahti, Emelie Stenman, Stefan Kauppinen, Anton Grundberg, Kristina Sundquist

**Affiliations:** ^1^ Center for Primary Health Care Research, Department of Clinical Sciences Malmö Lund University Malmö Sweden; ^2^ University Clinic Primary Care Skåne University Hospital Malmö Sweden; ^3^ Stockholm Sports Trauma Research Center, Department of Molecular Medicine and Surgery Karolinska Institutet Stockholm Sweden

**Keywords:** body checking, concussions, contact sport, female athlete, sport injuries

## Abstract

In 2022, the Swedish Women's Hockey League (SDHL) became the first women's league in the world to allow body checking. This study examines injury epidemiology in SDHL 2 years after this policy change. Injuries resulting in time loss from training or games were recorded in a digital injury‐reporting system. This study utilizes game‐related injuries that occurred during the seasons 2022–2023 and 2023–2024 in SDHL to examine injury epidemiology. Overall, 46 game‐related injuries were reported. The game injury rate (IR) was 17.2 (12.6–23.0) per 1000 player–hours. Body checking was the most common individual injury mechanism (*n* = 9; 20%) and concussions were the most frequent injury type, accounting for all head injuries (IR: 3.7 (1.8–6.9), *n* = 10; 22%). Most commonly, injuries occurred in the third period (*n* = 21; 46%), where nine of them occurred within the final six minutes of the game. Of all concussions (*n* = 10; 22%), six occurred in the third period. In 17 (37%) of all injuries and in five of 10 (50%) concussion events, the player continued to play despite sustaining an injury. This descriptive study is the first to examine injury epidemiology in elite women's ice hockey permitting body checking. Although concussions were the most common injury type, most commonly attributed to body checking, the results should be interpreted cautiously due to a limited number of injuries, estimated exposure, the absence of a comparator cohort, and incomplete reporting. The findings highlight the importance of injury surveillance strategies when introducing body checking in women's ice hockey.

## Highlights

- This is the first descriptive study examining injury epidemiology in elite women's ice hockey, following the introduction of body checking in the Swedish Women's Hockey League (SDHL).
- The overall game injury rate was 17.2 per 1000 player–game hours across the 2022–2023 and 2023–2024 seasons.
- Concussion was the most common injury type, most commonly attributed to body checking, which was the most commonly reported injury mechanism.
- Although based on a limited number of injuries, a substantial proportion of players continued playing after injury including after sustaining a concussion highlighting potential areas for improved injury and concussion recognition and surveillance.

## 1 Background

Ice hockey is a team contact sport known for its high intensity. Players can reach skating speeds up to 40 km/h (Flik et al. 2005), leading to high‐velocity collisions. Pucks can travel at speeds over 160 km/h (Flik et al. 2005), and players are equipped with sharp blades and sticks. The game is played on a hard surface of ice throughout a tightly scheduled game season. All these factors contribute to injuries from high‐energy trauma and overload.

The global rules for men's and women's ice hockey are similar with one important exception; body checking used to be prohibited for all levels of women's ice hockey. Body checks are typically performed by shoulder or hip contact with a player possessing the puck. Legal checks must be made within the rules, targeting the opponent's torso or hips, and cannot be aimed at the head, back, or below the knees. In non‐checking leagues, including most levels of women's and girls' hockey, body checking is penalized (International Ice Hockey Federation (IIHF) 2023).

Despite differences in body‐checking rules, collisions are the most common mechanism of injury and concussion the most common injury in both sexes, with higher concussion rates among women (Lynall et al. 2018; Wilcox et al. 2014; Kosziwka et al. 2021). In men's ice hockey, body checking is associated with higher rates of potentially serious injuries such as concussions, which not only cause suffering for the individual but also result in contact with healthcare services, time loss from sport, in some cases long‐term health implications (C. A. Emery et al. 2022; Macpherson et al. 2006). On the other hand, body checking is an important part of the game as it is deeply rooted in the sport's culture and history. Banning body checking in women's ice hockey raises concerns about gender inequality (Weaving and Roberts 2012), fueling a debate over whether it should be introduced among women.

In 2022, the Swedish Women's Hockey League (SDHL) was the first league in the world to allow body checking among females, creating a unique natural experiment to study injury epidemiology following the introduction in elite women's ice hockey. Even if female players themselves are positive toward introducing body checking (Lahti et al. 2024), no previous study has examined the injury epidemiology in women's elite ice hockey where body checking is allowed. A better understanding of injuries associated with body checking among female elite players will help decision‐making in other countries' women's leagues where body checking is still prohibited and aid the design of surveillance strategies.

This 2‐year prospective cohort study aims to examine the injury epidemiology in female elite ice hockey players 2 years after the introduction of body checking, including concussions and body checking injuries.

## 2 Method

This study uses data from the “Women's Ice Hockey Injury Study”, a research project with the broader aim of examining injuries and potential risk factors among female elite ice hockey players in SDHL. A detailed description of the study method has been presented in a previous study by the same research group (Lahti et al. 2024). The present study is conducted in adherence to the Declaration of Helsinki, approved by the Swedish Ethical Review Authority (Dnr: 2023‐06114‐01) and registered at ClinicalTrials.gov (ID: NCT06351618).

In 2022, the SDHL became the first league in the world to introduce body checking among women. The same year, a digitalized injury‐reporting system was implemented in the league where all teams are required by the Swedish Ice Hockey Association and the league SDHL to report injuries that lead to absence from training or a game. The diagnostics and registration of an injury into the injury‐reporting system by filling in an injury card is conducted by representatives from the medical team, including physicians, physiotherapists, and osteopaths. The injury card recorded a variety of injury details presented in Appendix 1.

Only injuries sustained during the official SDHL games during the 2022–2023 and 2023–2024 seasons were included in the present study. Although injuries sustained during training sessions, friendly games, and international tournaments were also recorded into the injury reporting system, the lack of exposure data from these events prevented the calculation of injury rates (IR) and were therefore not included in the statistical analysis of the present study (Stovitz and Shrier 2012; Donskov et al. 2019; Bahr et al. 2020).

In total, one injury occurred during international tournaments, and 15 during training sessions and were excluded from the analysis. Dental injuries and illnesses were not included as they were not reported into the injury reporting system. In addition, only time‐loss injuries were reported to the injury‐reporting system. “Time loss” refers to number of days lost from training or a game due to injuries, and these injuries are referred to as time‐loss injuries (Stovitz and Shrier 2012).

All injuries, including concussions, were diagnosed and recorded by members of each team's medical staff, including physicians, physiotherapists, and osteopaths, based on clinical assessment and according to standard concussion management practices. At baseline, all 225 players registered in SDHL 2023/2024 were invited to participate and received an email invitation. Of the 225 players invited at study start, 157 (70%) players agreed to participate in the research project (Lahti et al. 2024). Participation was voluntary, with the option to withdraw at any time. The included players represented all 10 teams in the league and all players' positions. However, in the initial season of registration during 2022–2023, only three teams within the league utilized the digitalized injury‐reporting system and by the 2023–2024 season, eight out of 10 teams in SDHL had adopted the register.

E‐mail addresses were provided by club and league representatives who facilitated the study but did not access or analyze any data, apart from each team's access to their own data. In the e‐mail, the players research information and a survey link via e‐mail, detailing the study, procedures, risks, benefits, and ethical considerations. Players who consent to participate were directly guided to the online survey collecting responses to a range of health‐related questions. In the present study, only the variables of age, body height, and body weight were extracted from the online survey and utilized in the present study. Body mass index (BMI) was calculated using the standard formula (kg/m^2^).

All other variables except age, body weight, body height, and BMI that were included in the present study were obtained from the injury card and the associated injury reporting system. These comprised injury mechanism, injury type, anatomic location, injury date, injury severity, player position, and game‐specific factors including penalization, rink location, home versus away game status, game period, time of injury, and the player's immediate response to injury.

An injury mechanism was assessed based on information provided in the injury card and included whether the injury involved voluntary player contact (i.e., body checking), non‐voluntary player contact (i.e., player collisions), contact with objects (i.e., stick or puck), and non‐direct mechanisms (i.e., fall, distortions, sprints, shots, passes, savings by goalkeepers, and overload). It was also noted whether the injury was a re‐injury or not.

Based on this information, each injury was classified into the most probable primary injury mechanism, categorized as body checking, player collision, board contact, contact with stick or puck, or non‐direct mechanisms. In cases where multiple contributing factors were present, such as player contact followed by impact with the boards, the most dominant mechanism was determined based on a structured evaluation of the injury card. If the primary injury mechanism could not be determined with sufficient certainty due to incomplete reporting on the injury card, these cases were classified as “unknown”. When no information at all was recorded and the variable field was entirely blank, the injury mechanism was classified as “missing”.

Injury type was categorized as concussion, contusion, distortion, fracture, luxation/subluxation, or strain. Anatomical location of injury was classified into four regions: head (including face and neck), upper extremity (shoulder, upper arm, elbow, forearm, wrist, and hand), lower extremity (hip, groin, thigh, knee, lower leg, ankle, and foot), back/chest (throat/cervical, spine, lumbar spine, sacrum, torso, and abdomen), and “other”.

Injury date referred to the calendar date (yyyy‐mm‐dd) on which the injury occurred. This variable was used to map the monthly distribution of injuries and identify the month with the highest injury incidence. Injury severity was classified according to time loss from sport, using the categories: minor (1–7 days), moderate (8–28 days), and severe (> 28 days) (Stovitz and Shrier 2012).

The player position was recorded as goalkeeper, defenseman, or forward. Game‐specific factors included whether the game was played at home or away, the period of the game (warm‐up, first, second, or third period), the time during a period when the injury occurred (0–6 minutes, 7–13 minutes, or 14–20 minutes within a match period), the rink location (offensive, defensive, or neutral zone), and whether the injury event was associated with a referee‐assigned penalty, reported as yes, no, or “do not know” (unknown).

The player's immediate response to injury was categorized into five predefined options: continued playing and completed the game; continued playing but eventually leaving the game; quit the game but later returned; quit the game immediately without returning; or “do not know” (unknown).

### 2.1 Statistical Analysis

All statistical analyses were done in R version 4.4.2 (R Core Team, 2024). Descriptive data are presented as mean values with standard deviations (SD) or numbers and percentages.

The IR per 1000 player–game hours was calculated assuming that both teams had five players and a goalkeeper playing on the rink for the whole game of 60 minutes (Donskov et al. 2019). In the 2022–2023 season, only three of 10 SDHL teams reported injuries. For matches involving only one team that used the injury reporting system, exposure was estimated based on 6 players; if both teams reported, exposure was based on 12 players.

In 2023–2024, eight of 10 teams used the reporting system. The same assumptions were applied: 12 players for matches between two reporting teams, and 6 players when only one team reported. In total, 2670 player–game hours were recorded across both seasons (672 hours during 2022–2023 and 1998 hours during 2023–2024) and game injuries were recorded across 298 games—98 played by three teams during the 2022–2023 season and 200 by eight teams during the 2023–2024 season. Possible overtime and penalties were not considered in the total exposure time. IRs were calculated using estimated player exposure based on standard assumptions, as individual player ice‐time data were not available. IRs are presented with a 95% confidence interval (95% CI).

IR (assuming 12 players) = (*n*° of injuries / (*n*° of games × 12 players × game duration)) × 1000.

If a player sustained two different injuries, each was recorded as a separate case in the injury dataset.

Given the relatively small number of injuries, analyses were limited to descriptive statistics, and no formal statistical comparisons between subgroups were performed.

## 3 Results

Descriptive statistics for the 46 injury occasions are presented in Table 1, with players counted once for each injury sustained. The largest proportion of injuries was sustained in forwards (*n* = 22; 48%), followed by defensemen (*n* = 18; 39%), and goalkeepers (*n* = 6; 13%). Findings from a previous dropout analysis showing no significant differences in age or body composition between participants and non‐participants, supporting the representativeness of the cohort (Lahti et al. 2024).

**TABLE 1:** Descriptive characteristics of the 35 players involved in the 46 injury occasions included in the present study, occurring in the Swedish Women's Hockey League (SDHL) during the 2022–2023 and 2023–2024 seasons. Weight and height were recorded during season 2023–2024. Continuous variables are presented as mean (SD) and player position as *n* (%).

Age (years)	23.8 (3.9)
Body weight (kg)	69.4 (6.3)
Body height (centimetres)	170.5 (5.6)
Body mass index (kg/m^2^)	23.9 (1.4)
Player position per injury, *n* (%)	
Forwards	22 (48)
Defensemen	18 (39)
Goalkeepers	6 (13)

The overall game IR was 17.2 (12.6–23.0) per 1000 player–game hours (IR: 16.4 (8.2–29.3) during the season 2022–2023 and 17.5 (12.2–24.4) during the season 2023–2024) (Table 2).

**TABLE 2:** Game injury rates (IR) across the entire study period and divided by different seasons.

	IR per 1000 game hours (95% CI)
All games	17.2 (12.6–23.0)
2022–2023	16.4 (8.2–29.3)
2023–2024	17.5 (12.2–24.4)

Among the 157 players who consented to participate, a total of 46 game‐related injuries were recorded in 35 players (22% of the study population). Twenty‐seven players had one recorded injury, and eight players recorded two or more injuries during the entire study period, spanning the two seasons 2022–2023 and 2023–2024 (data not shown).

Of the total 46 game‐related injuries, 11 occurred in the 2022–2023 season among the three teams who reported injuries during this season and 35 in the 2023–2024 season where eight out of 10 teams utilized the injury reporting system. Three of the game‐related injuries (6%) were re‐injuries that occurred within 6 months after the primary injury (data not shown).

Following injury, 59% of players (*n* = 27) quit the game immediately, whereas 37% (*n* = 17) continued playing despite the injury, with 5 of them eventually leaving the game and data were missing for this variable in two cases (4%). Two injuries (4%) resulted in a penalty assessed by the referee, 40 injuries (87%) did not, and in 4 cases (9%) this information was missing (data not shown).

Nearly half of all game injuries (*n* = 21; 46%) occurred during the third period, compared with 12 (26%) and 11 (24%) in the second and first periods, respectively. One injury (2%) occurred during warm‐up and for one injury (2%) data were missing. Notably, 17 (37%) game injuries took place during the final six minutes (minutes 14–20) of a period, where nine of them occurred during the third period. Regarding rink location, 23 (50%) of game injuries occurred in the defensive zone, 17 (37%) in the offensive zone, and 5 (11%) in the neutral zone, with missing data for 1 injury (2%). More injuries (*n* = 29; 63%) occurred during home games than away games (*n* = 16; 35%), with 1 injury (2%) missing (data not shown).

### 3.1 Reported Injury Mechanism

The most frequently reported injury mechanisms were non‐direct mechanisms (i.e., fall, distortions, sprints, shots, passes, savings by goalkeepers, and overload) (*n* = 11; 24%) followed by body checking (*n* = 9; 20%), player collisions (*n* = 8; 17%), and contact with the boards (*n* = 7; 15%). Contacts with puck or stick were less commonly reported (Table 3). A total of 9 out of 46 game injuries (20%) were associated with body checking. Of these, 3 injuries (33%) were classified as mild, 1 (11%) as moderate, and 3 (33%) as severe; 2 (22%) injuries were not classified due to missing values (Table 3).

**TABLE 3:** Reported injury mechanisms, frequency, and severity for all injuries in the 2022–2023 and 2023–2024 seasons. Data are presented as *n* (%) per column.

Reported injury mechanism	Number of injuries, *n* (%)	Severity, *n* (%)			
		Mild	Moderate	Severe	Missing
Board contact	7 (15)	2 (14)	1 (12)	1 (14)	3 (18)
Body checking	9 (20)	3 (21)	1 (12)	3 (43)	2 (12)
Non‐direct (including distorsions)	11 (24)	6 (43)	2 (25)	2 (29)	1 (6)
Player collision	8 (17)	1 (7)	3 (38)	1 (14)	3 (18)
Puck	1 (2)	1 (7)	0 (0)	0 (0)	0 (0)
Stick	1 (2)	0 (0)	1 (12)	0 (0)	0 (0)
Unknown	9 (20)	1 (7)	0 (0)	0 (0)	8 (47)
Total	46 (100)	14 (100)	8 (100)	7 (100)	17 (100)

**FIGURE 1:**
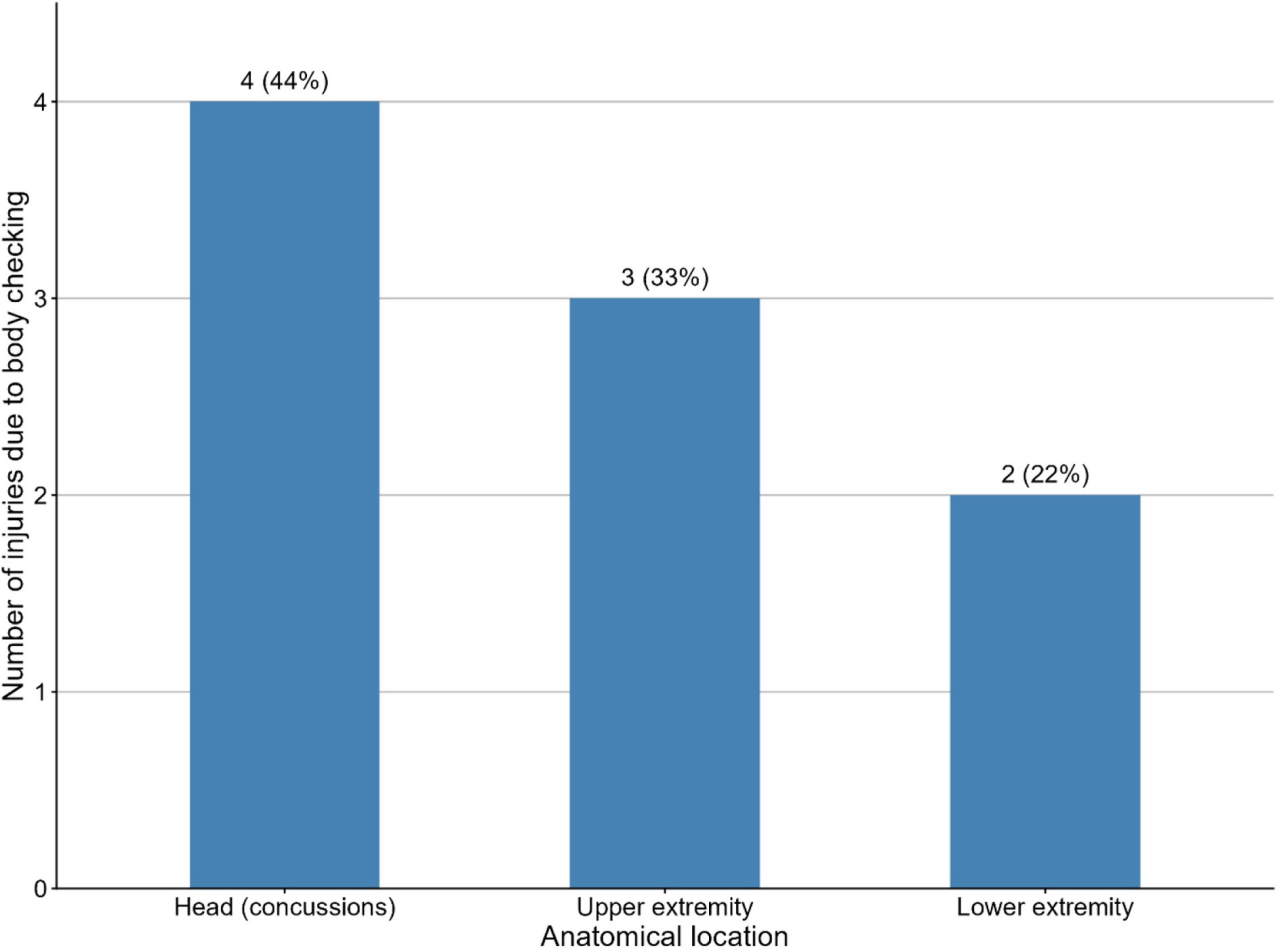
Injuries classified by anatomical location due to body checking in Swedish female elite ice hockey players during 2022–2024.

Six body checking injuries (67%) occurred in forwards and 3 (33%) in defensemen (data not shown). The head was the most injured anatomical location in body checking injuries (*n* = 4; 44%), followed by upper (*n* = 3; 33%) and lower extremity injuries (*n* = 2; 22%) (Figure 1). All head injuries were classified as concussions, which was also the most frequent injury type caused by body checking. The less frequent injury types caused by body checking were muscle strains, distortion, fractures, contusions, and luxation (data not shown).

In all 9 body checking injuries, the injured player was the recipient of the check; no injuries were reported among players delivering the body check. In 5 (56%) of the body checking injuries, the check was followed by board contact; in all other cases the check was on open ice, to the head or knee. Temporal patterns were similar to all injuries, with 4 (44%) of body checking injuries occurring during the third period. Notably, none of the body checking injuries resulted in a penalty. Most of the body checking injuries occurred in the offensive zone (*n* = 6; 67%), followed by the defensive zone (*n* = 2; 22%) and neutral zone (*n* = 1; 11%) (data not shown).

### 3.2 Injury Type

Among all 46 injuries recorded during the entire study period, concussions were the most frequently reported injury type (*n* = 10,22%), followed by distortions (*n* = 9.20%) and contusions (*n* = 7, 15%) (Table 4). Regarding concussions, 5 (50%) were classified as mild, 3 (30%) as moderate and none as severe; data were missing for 2 (20%) of the cases. Strains, fractures, overload, luxation, and lacerations were less common (Table 4).

**TABLE 4:** Injury types, incidence rates (IR) per 1000 player–game hours, and severity during the 2022–2023 and 2023–2024 seasons.

Injury type	Number of injuries, *n* (%)	IR per 1000 game hours (95% CI)	Severity, *n* (%)			
			Mild	Moderate	Severe	Missing
Concussion	10 (22)	3.7 (1.8–6.9)	5 (36)	3 (38)	0 (0)	2 (12)
Contusion	7 (15)	2.6 (1.1–5.4)	4 (29)	0 (0)	0 (0)	3 (18)
Distortion	9 (20)	3.4 (1.5–6.4)	2 (14)	1 (12)	2 (29)	4 (24)
Fracture	4 (9)	1.5 (0.4–3.8)	0 (0)	1 (12)	3 (43)	0 (0)
Luxation/subluxation	3 (7)	1.1 (0.2–3.3)	1 (7)	0 (0)	0 (0)	2 (12)
Strain	3 (7)	1.1 (0.2–3.3)	0 (0)	2 (25)	1 (14)	0 (0)
Unknown	10 (22)	3.7 (1.8–6.9)	2 (14)	1 (12)	1 (14)	6 (35)
Total	46 (100)	17.2 (12.6–23.0)	14 (100)	8 (100)	7 (100)	17 (100)

**FIGURE 2:**
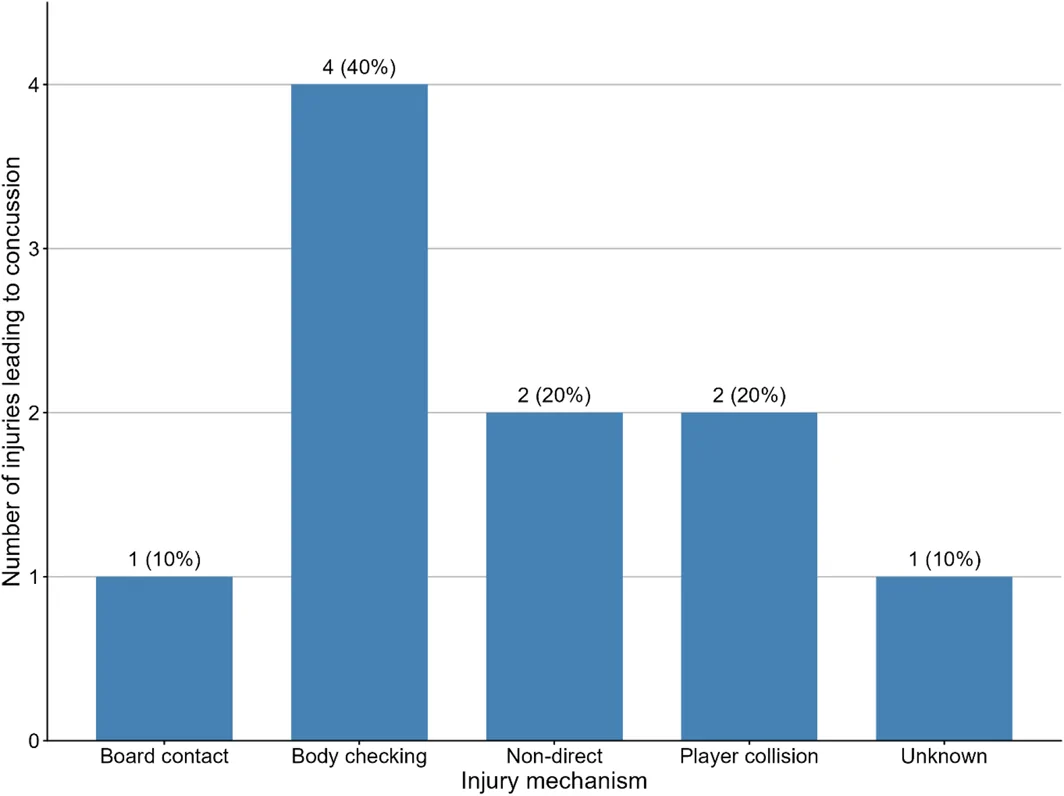
Reported injury mechanisms leading to concussion among female Swedish elite ice hockey players who are allowed body checking during 2022–2024.

The most common reported mechanism leading to concussion was body checking (*n* = 4; 40%), followed by collisions (*n* = 2; 20%) and non‐direct mechanisms (*n* = 2; 20%) while all other reported injury mechanisms were less frequent (Figure 2).

Half of the concussions (*n* = 5; 50%) were sustained by defensemen, followed by forwards (*n* = 4; 40%) and goalkeepers (*n* = 1; 10%). More than half of all concussions (*n* = 6, 60%) occurred in the third period, and 6 (60%) injuries took place in the final 6 minutes of the period. Of all concussions, five players (50%) discontinued play immediately by leaving the ice. In the other five cases (50%), the player initially continued playing despite the concussion; of these, two players subsequently withdrew from the game. Only one case (10%) of concussions resulted in a penalty, whereas the rest did not (data not shown).

### 3.3 Anatomical Location

The upper (*n* = 13; 28%) and lower extremities (*n* = 12; 26%) were the most frequently injured anatomic location (Table 5). However, the upper and lower extremity categories included a wide range of injuries, with the upper extremity covering the shoulder, upper arm, elbow, forearm, wrist, and hand, and the lower extremity including the hip, groin, thigh, knee, lower leg, ankle, and foot, whereas all head injuries were classified as concussions.

**TABLE 5:** Injury frequency, injury rates (IR) per 1000 player–game hours, and severity by anatomic location in the 2022–2023 and 2023–2024 seasons.

Anatomic location	Number of injuries, *n* (%)	IR (95% CI)	Injury severity, *n* (%)			
			Mild	Moderate	Severe	Missing
Head injury	10 (22)	3.7 (1.8–6.9)	5 (36)	3 (38)	0 (0)	2 (12)
Upper extremity	13 (28)	4.9 (2.6–8.3)	3 (21)	1 (12)	2 (29)	7 (41)
Lower extremity	12 (26)	4.5 (2.3–7.9)	2 (14)	3 (38)	5 (71)	2 (12)
Back	3 (7)	1.1 (0.2–3.3)	2 (14)	1 (12)	0 (0)	0 (0)
Missing	8 (17)	3.0 (1.3–5.9)	2 (14)	0 (0)	0 (0)	6 (35)
Total	46 (100)	17.2 (12.6–23.0)	14 (100)	8 (100)	7 (100)	17 (100)

### 3.4 Injury Incidence by Month

**FIGURE 3:**
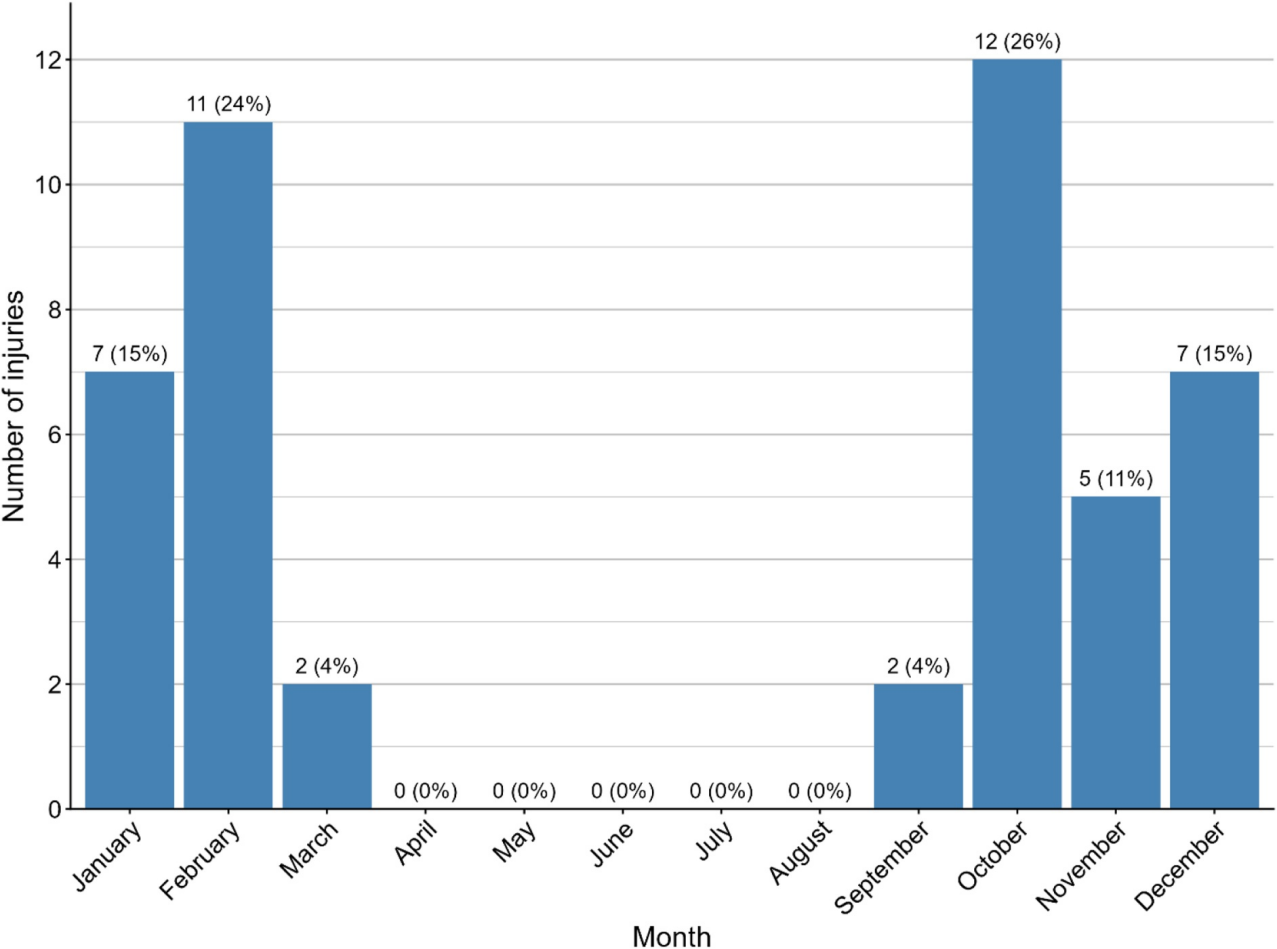
Seasonal variation in the number of game related injuries leading to time loss in female elite ice hockey players allowed body checking during 2022–2024.

Most game‐related injuries occurred in February and October, with October also showing the highest proportion of severe injuries resulting in more than 28 days of time loss (Table 6, Figure 3).

**TABLE 6:** Number of game‐related injuries and injury severity classified by month during the 2022–2023 and 2023–2024 seasons.

Month	Number of game injuries, *n* (%)	Severity, *n* (%)			
		Mild	Moderate	Severe	Missing
January	7 (15)	2 (14)	2 (25)	1 (14)	2 (12)
February	11 (24)	4 (29)	3 (38)	0 (0)	4 (24)
March	2 (4)	2 (14)	0 (0)	0 (0)	0 (0)
April	0 (0)	0 (0)	0 (0)	0 (0)	0 (0)
May	0 (0)	0 (0)	0 (0)	0 (0)	0 (0)
June	0 (0)	0 (0)	0 (0)	0 (0)	0 (0)
July	0 (0)	0 (0)	0 (0)	0 (0)	0 (0)
August	0 (0)	0 (0)	0 (0)	0 (0)	0 (0)
September	2 (4)	0 (0)	1 (12)	0 (0)	1 (6)
October	12 (26)	4 (29)	0 (0)	5 (71)	3 (18)
November	5 (11)	1 (7)	1 (12)	0 (0)	3 (18)
December	7 (15)	1 (7)	1 (12)	1 (14)	4 (24)
Total	46 (100)	14 (100)	8 (100)	7 (100)	17 (100)

## 4 Discussion

In this descriptive study of female elite ice hockey players, the overall injury IR was 17.2 (12.6–23.0) per 1000 player–game hours. Concussions, accounting for all head injuries, were the most frequently reported injury type, most commonly attributed to body checking. Despite important study limitations including a limited number of injuries, estimated exposure, absence of a comparator cohort, and incomplete reporting, this study provides a unique descriptive overview of injury patterns in a women's elite ice hockey league that permits body checking. The findings are hypothesis‐generating and provide a valuable foundation for future research in this understudied population.

Even though the category “non‐direct mechanisms” accounted for the largest proportion of injuries, this category comprised several heterogeneous mechanisms occurring too infrequently to be reported separately. When considered individually, body checking appeared to be the most common injury mechanism, most commonly associated with concussions. Furthermore, 43% of all reported body checking injuries were classified as severe.

A study of elite female ice hockey players in the Women's World Championship, where body checking is prohibited, reported an IR of 19.7 per 1000 player–game hours over eight years (Tuominen et al. 2016), broadly similar to that observed in the present study. However, methodological differences, including broader injury definitions, a longer observation period, and systematic reporting by the team doctor suggest that the present study may have underestimated the true IR.

Despite this, concussion rates were higher in the present study than in the Women's World Championships and the Olympic Winter Games, where body checking is prohibited (3.7 per 1000 game hours vs. 2.7 per 1000 game hours) (Tuominen et al. 2017). A review of sport‐related concussions across various sports, primarily involving U.S. high school and collegiate female athletes, reported the highest game‐related concussion incidence rates in rugby (8.2–16.1 per 1000 game hours) followed by Gaelic football (5.2 per 1000 game hours) (Ayrton et al. 2022). In Swedish female elite football, the concussion IR is 1.2 per 1000 game hours (Vedung et al. 2020) and in women's ice hockey, where body checking is still prohibited 2.7 per 1000 game hours (Tuominen et al. 2017). In comparison, the concussion IR observed in the present study was 3.7 (1.8–6.9) per 1000 game hours. However, comparisons between studies should be interpreted cautiously given differences in study design, injury definitions, surveillance methods, and exposure estimation.

Associations between body checking and sport‐related concussions have previously been described among male ice hockey players. For example, one study of male 15–17‐year‐old Canadian junior hockey players found that delaying the introduction of body checking was associated with a 70% reduction in overall IR and a 57% reduction in concussion IR (C. A. Emery et al. 2022). Similar trends have been found in male Canadian 13–15‐year‐olds (C. Emery et al. 2011, 2020). In addition, a recently published insurance‐based study by the same research group showed that the overall injury incidence rate nearly doubled following the introduction of body checking (Lahti et al. 2026).

The present study cannot determine if this policy change increased injury and concussion rates, or not. Before any causality can be established, this question requires further investigation in larger studies including pre‐ and post‐implementation periods and a comparator group. Notably, none of the body checking injuries in the present study resulted in penalties and all reported body checking injuries occurred among recipients of the check. Both observations are based on a small number of events and should be considered exploratory and need to be confirmed in future studies.

In the Swiss professional male ice hockey league, the overall IR has been as high as 88.6 per 1000 game hours (Brunner et al. 2020), in Swedish male elite players 74.1 per 1000 player–game hours (Pettersson et al.), in male adult Olympic game 52.1 per 1000 game hours (Tuominen et al. 2015), in the Finnish highest male elite league 47.4 per 1000 game hours (Hirvelä 2024) and in NHL 49.4 injuries per 1000 game hours (McKay et al. 2014). By comparison, the overall injury rate (IR) in the present study of female elite ice hockey players was 17.2 per 1000 player–game hours. Nevertheless, body checking was the most common injury mechanism in both sexes (Brunner et al. 2020; Tuominen et al. 2015; Hirvelä 2024; McKay et al. 2014; Suzuki et al. 2024). Compared to male NHL players, the present study on female players showed a higher concussion IR (3.7 vs. 1.8 concussions per 1000 game hours) (Benson et al. 2011), although comparisons between studies should, again, be made cautiously.

Half of all players who sustained a concussion, and more than one third who sustained any other kind of injury, continued to play. The reasons for continued participation after injury and concussion were not assessed in the present study and warrant further investigation in future studies.

In terms of concussion severity, 50% of concussions were classified as mild, 30% as moderate, and none as severe, although severity data were missing for two cases (20%). Most injuries, including concussions, occurred during the third period and within the final 6 minutes of a period. Injury frequencies were highest in February and October. In addition, more injuries were observed in the defensive zone, among forwards, and during home games. These observations are based on a small number of events and should be considered exploratory and require confirmation in larger studies.

Despite the biological differences between males and females, much of the existing concussion research is based on male athletes. A recent review found that only 19% of participants in major consensus statements were female, with 40% of studies excluding women entirely (D'Lauro et al. 2022). The present study contributes to the sparse literature on concussion and injury epidemiology in women's ice hockey.

### 4.1 Strengths and Limitations

Study strengths include the inclusion of all SDHL teams, 70% of players, and all player positions. Cohort representativeness is supported by a previous dropout analysis showing no differences in age or body composition between participants and non‐participants, and by similar anthropometric measures to those reported in the North American Professional Women's Hockey League (PWHL), enhancing the generalizability of the findings (Lahti et al. 2024).

Another strength is that all injuries were diagnosed and reported by team medical staff, increasing the likelihood of accurate injury identification and classification. The study findings also provide a valuable foundation for future research in this understudied population that could, if confirmed, provide valuable insights for global policy decisions on body checking in women's ice hockey.

The study also has several limitations, including a small sample size, few recorded injuries, and a short follow‐up period of only 2 years after the introduction of body checking. Consequently, statistical power was limited, and findings may have been influenced by a random variation. The lack of player–level ice‐time data limits the precise exposure and the absence of a comparator cohort from a non‐body‐checking league limits causal interpretation. Missing data for several game‐specific variables, particularly injury mechanisms, penalization, and in‐game context, may also have influenced some results. In addition, the present study also has fewer recorded injuries compared to other studies (Tuominen et al. 2016, 2017, 2015). Long‐term surveillance is needed to strengthen conclusions and statistical reliability as well as enabling subgroup analysis by player position and age.

Another limitation is the incomplete and varying reporting coverage between seasons, which may have resulted in underestimation of the number of injuries. Differences in concussion awareness, medical team composition and resources, diagnostic practices, and injury mechanism classification may also have influenced the findings. Injury mechanisms were classified by medical personnel rather than referees, and some overlap and misclassification between categories, particularly body checking, board contact, and player collisions, cannot be excluded.

The exposure time was calculated based on the assumption that six or 12 players were always on the ice, effectively treating participation as 100%. Furthermore, not only team physicians like in other studies (Tuominen et al. 2016, 2017, 2015) but a variety of professionals within the medical teams diagnosed injuries, possibly affecting the diagnostic accuracy. Although the injury definition was consistent with the International Olympic Committee consensus statement, including injuries requiring medical attention or resulting in time loss from sport (Bahr et al. 2020), injuries not resulting in time loss may have gone unreported, potentially leading to an underestimation of the overall injury burden. In addition, exposure estimates did not account for overtime or penalties. Although return‐to‐play decisions in SDHL generally follow established guidelines, variation between teams cannot be excluded.

## 5 Conclusions

This descriptive study provides an observational overview of injury patterns in elite women's ice hockey following the introduction of body checking. Concussion was the most frequently reported injury type, most commonly attributed to body checking. The findings should be interpreted cautiously given the limited number of injuries, estimated exposure, absence of a comparator cohort, and incomplete reporting coverage. The results are exploratory but could, if confirmed in future larger studies, provide a basis for injury surveillance strategies when introducing body checking in women's ice hockey. Potential preventive approaches in the future may then include improved concussion recognition, education in safe checking techniques and fair play, consistent rule enforcement, and adequate recovery.

## Funding

Dr Per Håkanssons Stiftelse (10.13039/501100009727).

## Conflicts of Interest

The authors declare no conflicts of interest.

## Data Availability

The data that support the findings of this study are available on request from the corresponding author. The data are not publicly available because of privacy or ethical restrictions.

## Appendix 1

**Table:**

Injury card				
Injury date (yyyy‐mm‐dd)				
Date of return to full activity (yyy‐mm‐dd)				
Number of days of time loss				
Diagnosis (ICD10)				
Injury mechanism				
	Non‐direct			
		Fall		
		Distortion		
		Sprint		
		Shot		
		Pass		
		Saving (goalkeeper)		
		Other		
	Voluntary player contact			
		Body checking		
			Received	
			Given	
				Open ice
				Checking to the board
				Checking to the head
				Checking from the blind side/behind
				Towards abdomen
				Towards knee
		Other voluntary player contact		
			Elbow checking	
			Slashing	
			Cross checking	
			High stick	
			Slew foot	
			Other	
		Fighting		
		Unknown		
	Non‐voluntary player contact			
		Collision		
			Direct (with player or object). The contact leads directly to injury	
			Non‐direct (with player of object). The contact is a part of an injury chain that indirectly lead to an injury	
	Contact with object			
		Stick		
		Puck		
		Skate		
		Shoulder equipment		
		Other		
		Board		
		Ice		
		Goal		
		Other		
	Overload			
	Other			
Injury type				
	Acute			
		Distorsion		
		Luxation		
		Subluxation		
		Contusion		
		Wound injury		
		Fracture		
	Overload			
Anatomic location				
	Head/face			
	Throat/cervical spine			
	Shoulder			
	Upper arm Forearm			
	Elbow			
	Wrist			
	Hand			
	Torso			
	Lumbar Spine/Sacrum			
	Abdomen			
	Hip/groin			
	Thigh			
	Knee			
	Lower leg			
	Ankle			
	Foot			
	Other			
Re‐injury				
	Yes			
	No			
		If yes, how long time since the original injury		
			1–2 months	
			2–3 months	
			3–6 months	
			6–12 months	
			> 12 months	
The player’s immediate response to injury				
	Continued playing and completed the game			
	Continued playing but eventually leaving the game			
	Quit the game but later returned			
	Quit the game immediately without returning			
	Unknown			
Period during match				
	Warm‐up			
	Period 1			
	Period 2			
	Period 3			
Time during period				
	Beginning (0–6 minutes)			
	Middle (7–13 minutes)			
	End (14–20 minutes)			
	Unknown			
Game				
	Home			
	Away			
Place in the rink				
	Defence zone			
		Along the board		
		Behind the goal line		
		Central, in front of the goal		
		Central, close to the blue line		
	Neutral Zone			
	Offensive zone			
		Along the board		
		Behind the goal line		
		Central, in front of the goal		
		Central, close to the blue line		
Penalization by the referee				
	Yes			
	No			
	Do not know			
Player position				
	Goalkeeper			
	Defencemen			
	Forward			
